# Perioperative alpha blockers in voiding dysfunction secondary to prostate biopsy: A meta‐analysis

**DOI:** 10.1002/bco2.366

**Published:** 2024-05-08

**Authors:** Sean Lim, Kylie Yen‐Yi Lim, Liang Qu, Sanjeeva Ranasinha, Anthony Dat, Matthew Brown, Paul Manohar, Matthew Harper, Scott Donnellan, Weranja Ranasinghe

**Affiliations:** ^1^ Department of Urology Monash Health Melbourne Victoria Australia; ^2^ Department of Medicine, Nursing and Health Sciences Monash University Clayton Victoria Australia; ^3^ Department of Public Health and Preventive Medicine Monash University Clayton Victoria Australia; ^4^ Department of Urology Fiona Stanley Hospital Murdoch Western Australia Australia

**Keywords:** alpha blockers, LUTS, prostate biopsy, prostate cancer, voiding dysfunction

## Abstract

**Introduction and Objectives:**

Voiding dysfunction remains a common side effect postprostate biopsy leading to significant morbidity. Alpha blockers have emerged as a potential therapeutic option to mitigate this risk, with various centres already incorporating its use in practice. Despite this, the literature regarding its efficacy remains inconclusive. Hence, a systematic review was performed to quantify the effect of perioperative alpha blockers on prostate biopsy‐related voiding function.

**Methods:**

A systematic search in MEDLINE, Embase and PubMed between January 1989 and July 2023 was performed to identify relevant articles. Two independent reviewers independently screened abstracts, full texts and performed data extraction. Data including International Prostate Symptom Scores (IPSS), voiding flow rates (Qmax), postvoid residuals (PVR), rates of acute urinary retention (AUR) and quality of life (QoL) scores were extracted. Results were combined in an inverse variance random effects meta‐analysis.

**Results:**

A total 808 patients from six randomised controlled trials (RCTs) comparing alpha blockers to controls were included. All articles excluded patients with pre‐existing voiding dysfunction. Pooled outcomes demonstrated statistically significant differences favouring alpha blocker usage in all objective and subjective measures including IPSS (mean difference 4.21, 95% confidence interval [CI] 2.58–5.84, *p <* 0.00001), PVR (mean difference 20.41 mL, 95% CI 3.44–37.39, *p =* 0.02), Qmax (mean difference 3.07 mL/s, 95% CI 2.55–3.59, *p <* 0.00001), QoL (weighted‐mean difference 0.82, CI 0.17–1.48, *p =* 0.01) as well as overall risk of AUR (odds ratio 0.22, CI 0.09–0.55, *p =* 0.001). There was variable heterogeneity (*I* ^2^ = 0–86%) between outcomes.

**Conclusions:**

This review highlights the potential role of alpha blockers in improving urinary function and reducing adverse voiding outcomes postprostate biopsy. The standard practice of incorporating the usage of perioperative alpha blockers may be considered to reduce the morbidity of voiding complications secondary to prostate biopsy.

## 1 INTRODUCTION

Prostate cancer constitutes a significant health burden worldwide, as the most common solid organ malignancy in men. The diagnosis and management of prostate cancer are based on histologic ascertainment of the tumour using prostate biopsy. As such, approximately one million prostate biopsies are performed each year in the United States alone.^1^

Yet, the benefits of prostate biopsies need to outweigh its associated morbidity. With the advancement of technology and innovation, prostate biopsies are currently performed using transrectal (TRUS) or transperineal (TP) approaches. Both methods have been shown to be relatively safe but are also associated with complications. However, current European Association of Urology (EAU) guidelines recommend the usage of TP over TRUS biopsies due to an increased risk of infectious complications secondary to the TRUS approach.^2^ Of the noninfectious complications following prostate biopsy, voiding dysfunction (characterised by urinary difficulties such as decreased flow, urinary retention, urinary frequency, urgency, or incomplete emptying) remains most prevalent occurring in up to 51.5% of patients.^3^, ^4^, ^5^, ^6^ As these symptoms can lead to significant distress and affect patients' quality of life (QoL), identifying those at risk and minimisation of these complications is critical.

Addressing voiding dysfunction after prostate biopsy requires a thorough understanding of its underlying mechanisms and potential treatment strategies. Alpha‐1 blockers result in relaxation of smooth muscle of the prostate enabling the passage of urine more freely through the prostatic urethra, making this a useful medication for prostate‐related voiding dysfunction affecting patients with benign prostate hyperplasia (BPH) and biopsy‐related prostatic inflammation. A meta‐analysis demonstrated that the usage of alpha‐1 adrenergic agents may have a role in prevention of postoperative voiding dysfunction and urinary retention.^7^ Among the promising interventions, alpha blockers such as Tamsulosin, Silodosin and Doxasozin have emerged as a potential therapeutic option to alleviate voiding symptoms postbiopsy.^8^, ^9^ At present, various health services such as Western Health in Australia^10^ and West Suffolk NHS Foundation Trust in the United Kingdom^11^ incorporate the standard practice of administering pre‐operative and postoperative alpha blockers for all patients undergoing prostatic biopsy.

Despite the growing interest in the usage of alpha blockers for managing voiding dysfunction after prostate biopsy, there is limited literature and therefore inconclusive evidence on this topic. We conducted a systematic review of published studies to assess the impact of alpha blockers on voiding function following prostate biopsy.

## 2 METHODS

**FIGURE 1:**
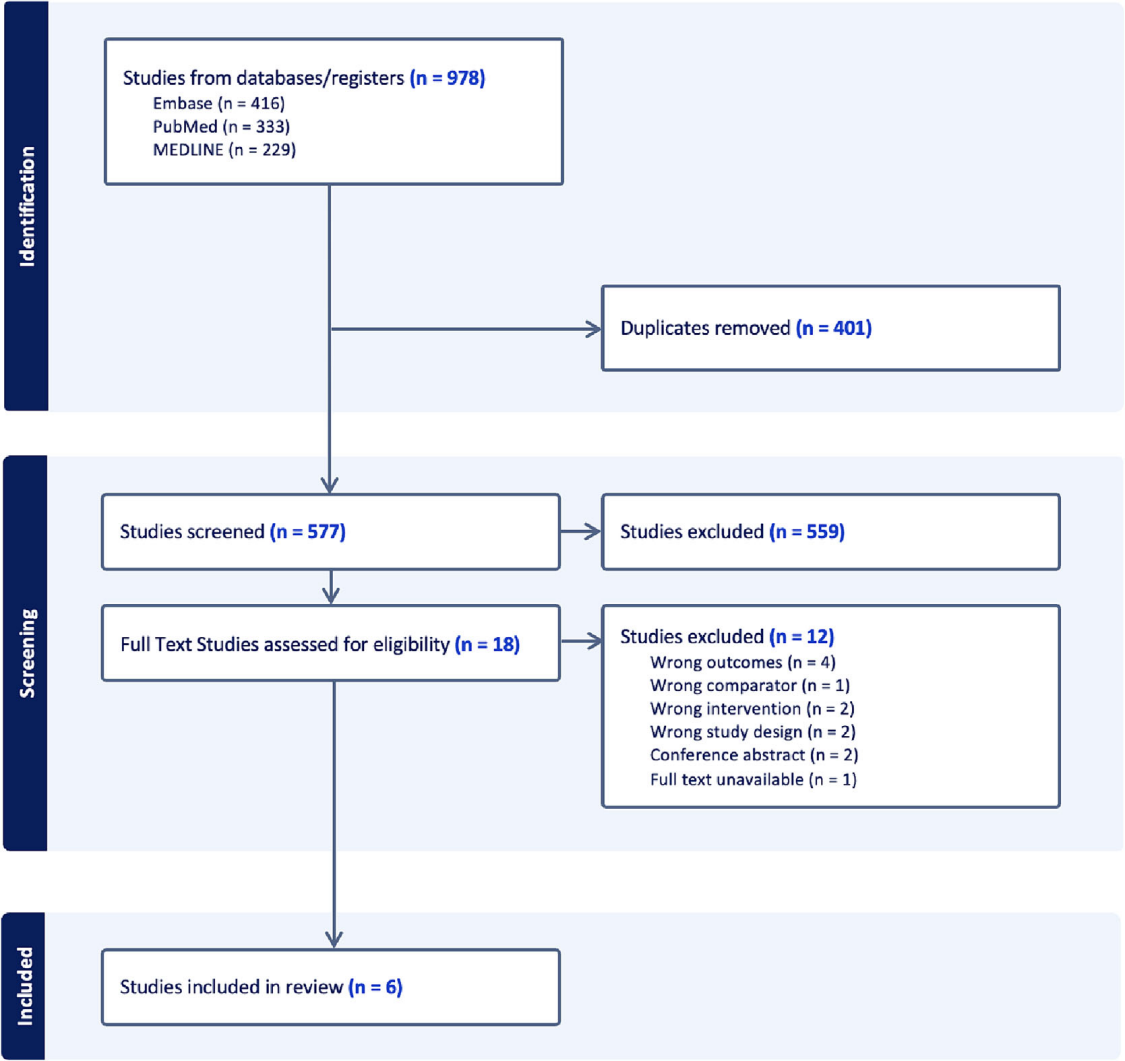
Preferred Reporting Items for Systematic Reviews and Meta‐Analyses (PRISMA) flow chart of included and excluded studies.

This systematic review is registered with the International prospective register of systematic reviews (PROSPERO ID ‐ CRD42023448173) and conducted in accordance with the Preferred Reporting Items for Systematic Reviews and Meta‐Analyses (PRISMA) Guidelines.^12^ Review methods were established prior to conduct, and no deviations occurred (Figure 1).

### 2.1 Search strategy

MEDLINE, Embase and Pubmed databases were searched from 1989 to July 2023. Medical subject headings and keywords for ‘alpha blocker’, ‘prostate’ and ‘biopsy’ were combined with Boolean operators to identify potentially relevant articles. The search strategy is outlined in supporting information Table S1. A sensitive search strategy was employed to identify articles investigating the usage of alpha blockers during prostate biopsy.

### 2.2 Study selection

Studies were included if they assessed the usage of perioperative alpha blockers during prostate biopsy and their effect on postbiopsy voiding function. Studies were excluded if (1) outcome assessed did not include measurements of voiding function, (2) population did not undergo prostate biopsy, (3) alpha blockers were not administered perioperatively, (4) population had preexisting conditions affecting urinary function, (5) it was a duplicate study, (6) it was a non‐English study and (7) manuscript was not available for review or only available in abstract form with limited data. Both observational (prospective, retrospective cohort studies) and interventional (randomised controlled trials [RCTs]) study designs were included if satisfied inclusion and exclusion criteria.

Any method of prostate biopsy was included, such as TP or TRUS biopsies. All alpha blockers, including Tamsulosin, Prazosin, Doxazosin, Alfuzosin and Silodosin were included regardless of dosing or duration as long as this included any period intra‐operatively or postoperatively.

Measurements of voiding function included subjective measurements such as International Prostate Symptom Scores (IPSS), QoL or objective measurements including maximum voiding flow rates (Qmax) and postvoid residuals (PVR) as well as incidences of acute urinary retention AUR or voiding dysfunction.

Two reviewers (SL and YL) independently screened titles and abstracts to identify relevant articles. The full texts of relevant articles were then evaluated against the strict inclusion and exclusion criteria. Disagreements between the reviewers were resolved by consensus.

### 2.3 Data extraction

Data extracted by two independent reviewers (SL and YL) were type and location of study, year of publication, patient characteristics, mean prostate volumes (PV), serum prostate‐specific antigen (PSA) levels, biopsy method, inclusion and exclusion criteria for patient enrolment and biopsy, alpha blocker type, dose and duration, additional interventions and outcomes including timing of measurements. Specific subjective and objective outcomes measured included IPSS (including voiding and storage scores if provided), QoL, Qmax, PVR and rates of AUR. All measurements at any timeframe recorded by studies were extracted including prebiopsy and postbiopsy results. Values including means, medians, ranges, interquartile ranges, standard deviations, standard errors and raw values were recorded if provided.

### 2.4 Quality assessment

Quality assessment was performed by two independent blinded authors (SL, YL) using the Cochrane Risk of Bias Tool Version 2 (RoB 2).^13^ The Cochrane RoB 2 tool includes the following domains: selection bias (random sequence generation, allocation concealment), performance bias (blinding of participants), detection bias (blinding of clinicians), attrition bias (loss to follow‐up) and reporting bias (selective reporting). All conflicts were resolved by discussion.

### 2.5 Data synthesis and statistical analysis

Inverse variance, random‐effects meta‐analyses were performed to assess pooled effects of interventions on individual outcomes. Random‐effects meta‐analyses using a DerSimonian and Laird estimator based on inverse variance weights were employed.^14^ Random‐effects meta‐analysis was chosen, as heterogeneity was anticipated because of between‐study variations in clinical factors between studies such as different alpha blockers, timings of application or patient characteristics.

For the dichotomous outcome (AUR), summaries were produced for each study by calculating the ORs and 95% confidence intervals (CIs). Continuous outcomes (IPSS, PVR, Qmax and QOL) were expressed as differences in standardised means between interventions and controls calculated with 95% CIs in pooled data.

The presence or absence of heterogeneity was determined by the chi‐square test, and the magnitude of heterogeneity was assessed by the *I* ^2^ statistic.^15^ Heterogeneity was considered present when the chi‐square test revealed a *p <* 0.05, and magnitude of heterogeneity was considered low, moderate or high with an *I* ^2^ statistic of <25%, 25%–75% and >75%, respectively. A meta‐regression analysis was performed to investigate sources of heterogeneity, including mean age, PSA, PV and treatment (alpha blockers). Publication bias was assessed by visual inspection of funnel plots and applying Egger's test set at a threshold of a *p*‐value less than 0.05 to indicate funnel plot asymmetry.^16^ Statistical significance was defined as two‐sided *P <* 0.05.

Statistical analysis was conducted on STATA (Stata Corp LP. Version 16.0. STATA/IC for Windows. College Station, Texas).

## 3 RESULTS

The electronic search yielded 978 results (Figure S1). After removal of duplicates, title/abstract screening and full text screening, six studies were included for analysis.^3^, ^8^, ^9^, ^17^, ^18^, ^19^ Articles were excluded during full text review for various reasons such as incorrect outcomes measured (*n =* 4), incorrect intervention/comparator groups (*n =* 3), incorrect study design (*n =* 3), available only in abstract form with limited data (*n =* 2) or full text unavailable (*n =* 1). One article was excluded as it compared the usage of Doxazosin to Doxazosin with Celecoxib rather than a control group.^20^

### 3.1 Quality assessment

**FIGURE 2:**
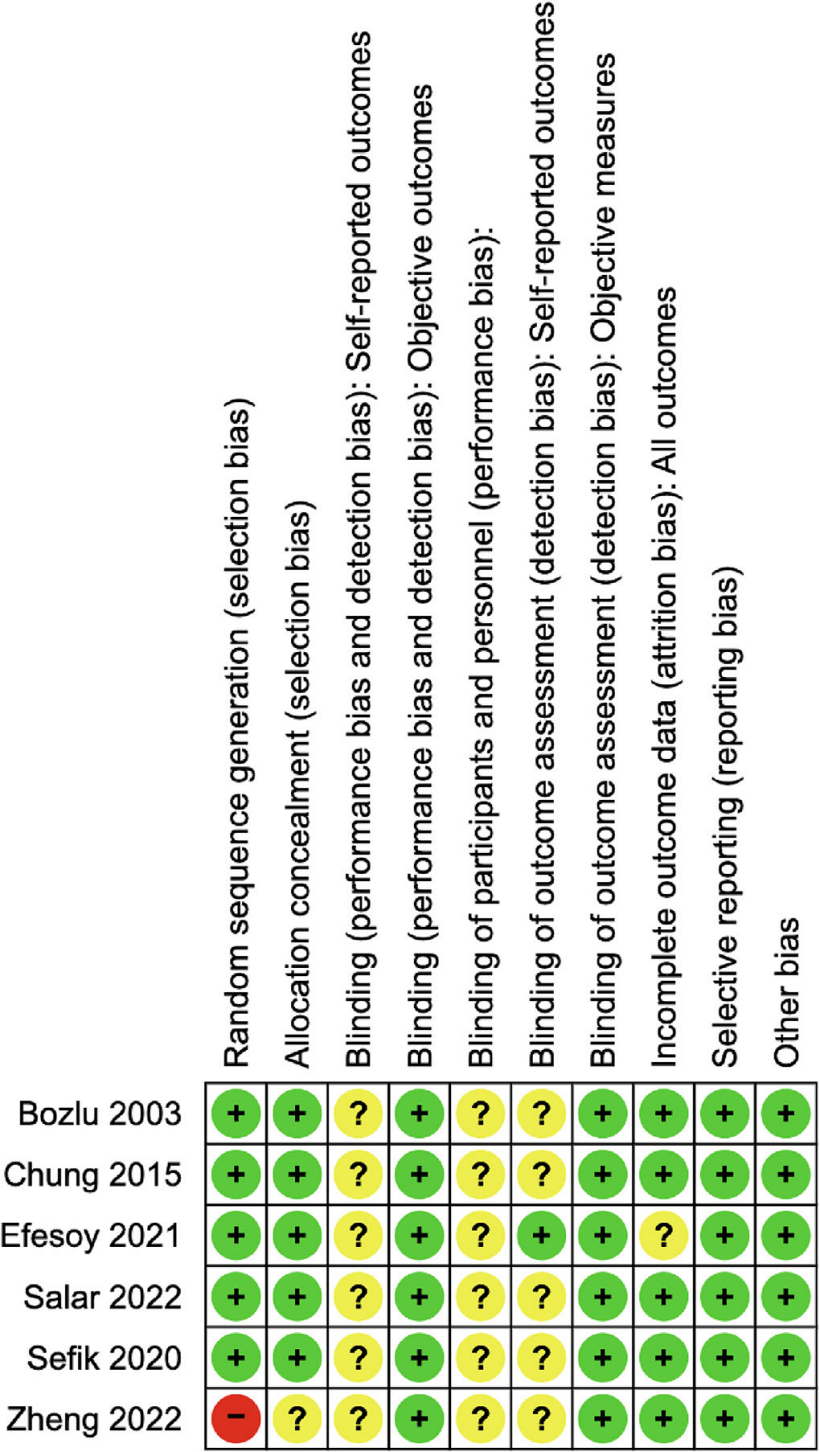
Risk of bias across Cochrane RoB‐2 criterion domains.

Results from quality assessment are summarised in Figure 2. Across articles, there was generally low risk of bias across included studies for selection bias, performance/detection bias for objective outcomes, attrition bias and reporting bias. However, there was unclear risk of bias for performance and detection bias for patient or clinician reported outcomes across studies due to. Sensitivity analysis requiring exclusion of poor quality studies was not performed as trials were similar in quality.

### 3.2 Study characteristics

The six studies conducted from 1989 to 2023 consisted of a total of 808 patients (Table 1). One study reported financial support by their national health science foundation,^18^ but the other five trials had no funding or conflicts of interest to declare. All studies were RCTs, comparing an intervention group of patients receiving perioperative alpha blockers during TRUS prostate biopsy to a control group of patients receiving no therapy. Sample sizes of each study varied from 66 to 200 patients. Indication for biopsy across all studies was elevated PSA values or abnormal digital rectal examinations (DRE). One study included patients with abnormal imaging findings including prostate MRI or ultrasound.^18^

**TABLE 1:** Characteristics of included studies.

Study	Study type	Indication for biopsy	Biopsy Method	Exclusion criteria	Sample size (alpha blocker/control)	Alpha Blocker	Alpha blocker duration and dose	Outcomes	Timing of Outcome Measure
Sefik (2020)^18^	RCT	PSA > 3 ng/mL OR Abnormal DRE	TRUS	Prior prostate biopsy, medical therapy for benign prostatic hyperplasia, history of prostate surgery, severe diabetes mellitus, severe coagulation disorders, rectal disease, concomitant malignancy, neurological diseases.	55/56	Tamsulosin	0.4 mg daily 1 week prior to 1 week post	IPSS, Qmax, PVR, QoL (SF‐36, Turkish Version),^21^ AUR	Baseline, day 7, day 30
Chung (2015)^17^	RCT	Serum PSA > 3 ng/mL OR Abnormal DRE	TRUS	History of AUR, medical/surgical treatment for benign prostatic hyperplasia, neurologic disease, bleeding diathesis or anticoagulation, urinary yract infection, no follow‐up data.	44/44	Tamsulosin	0.2 mg day prior for 7 days total	IPSS (Voiding/Storage), Qmax, PVR, QoL (IPSS), AUR	Baseline, day 1 (excluding IPSS), day 7
Zheng (2022)^18^	RCT	Elevated serum PSA OR abnormal DRE/Ultrasound/Magnetic Resonance Imaging	TRUS	History of prior prostate biopsy, AUR, diabetes mellitus, neurologic disease and bleeding diathesis.	100/100	Doxazosin	4 mg day prior to 30 days post	IPSS (Voiding/Storage), Qmax, PVR, QoL (IPSS), AUR	Baseline, day 7, day 30
Salar (2022)^8^	RCT	Serum 7PSA > 2.5 ng/mL OR abnormal DRE	TRUS	Urinary tract infection, urethral stricture, rectal disease, severe coagulopathy/diabetes mellitus/coagulopathy/neurological disease, prior of alpha blocker usage, prior prostate surgery/biopsy, prior AUR.	99/98	Silodosin	8 mg 10 days prior to 10 days post	IPSS, Qmax, PVR, AUR	Baseline, day 7
Bozlu (2003)^3^	RCT	Serum PSA > 4 ng/mL OR abnormal DRE	TRUS	Prior TRUS prostate biopsy, AUR, Medical or surgical treatment for benign prostatic hyperplasia, diabetes mellitus, neurologic disease, bleeding diathesis, current anticoagulation therapy, suspected urinary tract infection.	33/33	Tamsulosin	0.4 mg day prior to 30 days post	IPSS, Qmax, QoL (IPSS), AUR	Baseline, day 7, day 30
Efesoy (2021)^19^	RCT	Elevated/abnormal serum PSA or PSA derivatives OR abnormal DRE	TRUS	Diabetes mellitus, neurologic disease, bleeding diathesis, recent anticoagulation, genitourinary infections, PV < 40 mL, prior medical or surgical treatment of benign prostatic hyperplasia, urethral instrumentation/surgery, prior AUR, prior TRUS prostate biopsy.	121/25	Alfuzosin, Doxazosin, Silodosin, Tamsulosin, Terazosin	10 mg, 4 mg, 8 mg, 0.4 mg, 5 mg respectively day prior to 30 days post	IPSS, Qmax, PVR, QoL (IPSS), AUR	Baseline, day 7, day 30

Abbreviations: AUR, acute urinary retention; IPSS, International Prostate Symptom Score; PSA, prostate‐specific antigen; PVR, postvoid residual; RCT, randomised controlled trial; TRUS, transrectal; Qmax, voiding flow rates; QoL, quality of life.

Criteria for participant exclusion were similar across studies, such as prebiopsy voiding dysfunction, BPH or treatment of BPH, prior prostate biopsy, diabetes mellitus, rectal disease, neurological disease and coagulopathy. An exception to this was Efesoy 2021, which excluded patients with PV of <40 mL. Despite this, PV were noted to be similar across articles with a mean difference of 0 mL (*p =* 1.00) on pooled analysis.

A variety of alpha blockers were investigated between studies including Alfuzosin (*n =* 1), Doxazosin (*n =* 2), Silodosin (*n =* 2), Tamsulosin (*n =* 4) and Terazosin (*n =* 1). Daily dose administered to study participants were 10, 4, 8, 0.4 and 5 mg, respectively, for each alpha blocker. The exception was Chung 2015 which administered 0.2 mg of Tamsulosin following the standard of practice in South Korea. Alpha blockers were administered at various intervals from up to 10 days prior to biopsy until up to 30 days post (Table 1).

Sufficient data for meta‐analysis were available for all outcome measures of interest. All six included trials investigated postbiopsy IPSS, Qmax and AUR as outcomes with a pooled total of 808 patients. Five trials investigated PVR volumes and QoL scores as outcomes which pooled a total of 742 and 611 patients for each outcome, respectively. Of these, four trials utilised the QoL component of the IPSS questionnaire for measurement, and one trial utilised the Turkish version of the 36 Item Short Form Survey (SF‐36) for QoL assessment.^9^ Statistical measurement units for continuous outcomes were consistent across all articles (means and standard deviations or standard errors). Continuous outcomes were measured at various timings across studies including day 1, 7 and 30 postbiopsy, with all studies including day 7 for outcome measurements. Occurrences of AUR were recorded for patients at any point in time postbiopsy.

### 3.3 Patient characteristics

There were no differences noted in the distribution of baseline patient characteristics between intervention and control groups in each individual included study (Table 2) including age, PSA and PV. Additionally, pre‐op IPSS, Qmax, PVR and QoL scores were not statistically different between groups. Notably, Efesoy 2021 had a higher mean PV of 61.15 mL compared with other studies (39.0–53.52 mL) likely due to exclusion of patients with PV under 40 mL.

**TABLE 2:** Baseline patient characteristics in included studies.

Study	Intervention group	Sample size (*n*)	Age (year)	PSA (ng/mL)	PVol (mL)
Bozlu (2003)	Tamsulosin	33	63.56 ± 7.08	9.56 ± 8.12	49.65 ± 18.12
	Control	33	61.46 ± 6.93	8.31 ± 13.76	42.16 ± 18.14
Chung (2015)	Tamsulosin	44	66.1 ± 9.2	11.1 ± 15.8	40.6 ± 16.1
	Control	44	66.5 ± 7.4	34.8 ± 151.4	39.0 ± 16.3
Efesoy (2021)	Alfuzosin	24	59.79 ± 6.84	12.35 ± 13.46	59.04 ± 15.99
	Doxazosin	25	62.32 ± 6.98	13.06 ± 17.15	58.36 ± 17.32
	Silodosin	23	61.56 ± 8.57	13.91 ± 15.33	57.39 ± 22.01
	Tamsulosin	26	60.84 ± 6.85	12.16 ± 11.10	61.15 ± 25.50
	Terazosin	23	62.65 ± 5.36	11.64 ± 12.67	58.08 ± 17.72
	Control	25	61.96 ± 5.43	11.20 ± 15.36	54.35 ± 14.49
Salar (2022)	Silodosin	99	62 ± 7.4	8.1 ± 5.7	41.8 ± 11.1
	Control	98	63.2 ± 8.2	9.8 ± 6.1	45.4 ± 12.6
Sefik (2020)	Tamsulosin	55	63.6 ± 6.4	10.1 ± 6.7	56.8 ± 22.6
	Control	56	63.6 ± 6.4	8.2 ± 6.7	50.3 ± 17.5
Zheng (2022)	Doxazosin	100	68.4 ± 0.8^a^	17.2 ± 4.1^a^	38.8 ± 1.8^a^
	Control	100	67.2 ± 0.9^a^	24.2 ± 5.8^a^	39.3 ± 1.7^a^

*Note*: Values are represented as mean ± standard deviation.

^a^ Mean ± standard error.

### 3.4 IPSS

**FIGURE 3:**
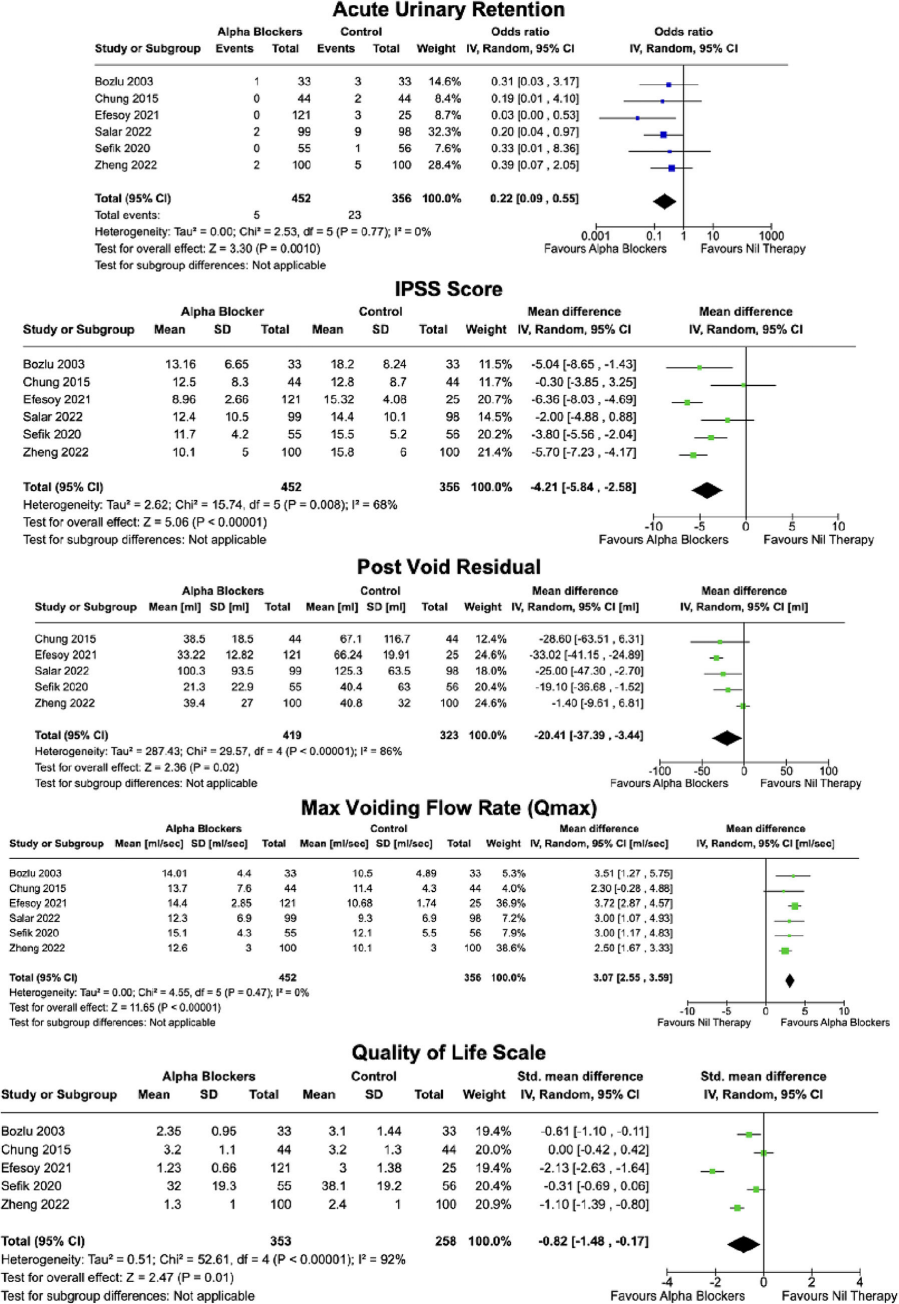
Meta‐analysis of all outcomes*. *Acute urinary retention, IPSS, postvoid residuals, Mox Voiding Flow rate, Quality of Life scales.

All six RCTs demonstrated lower IPSS at day 1, 7 and 30 postprostate biopsy for alpha blocker treatment groups compared with controls (Table 3). Four RCTs demonstrated statistically significant differences at day 7. Of the two studies not demonstrating statistical significance, one used a lower dosage of Tamsulosin (0.2 mg daily),^17^ and another used Silodosin as an intervention.^8^ Pooled analysis showed statistically significantly lower IPSS (mean difference 4.21, 95% CI: 2.58–5.84) in alpha blocker groups with moderate to high heterogeneity (*I* ^2^ = 68%) (Figure 3).

**TABLE 3:** Baseline and post‐biopsy IPSS, Qmax, PVR, QoL scores and AUR rates.

Study	Intervention group	Sample size (*n*)	IPSS		Qmax		PVR		QoL		AUR (*n*)
			Baseline	Post	Baseline	Post	Baseline	Post	Baseline	Post	
Bozlu (2003)	Tamsulosin	33	15.4 ± 6.78	Day 7 **13.16 ± 6.65** * Day 30 **10.77 ± 6.27** *	12.78 ± 5.44	Day 7 **14.01 ± 4.4** * Day 30 **15.6 ± 4.53** *	45.2 ± 21.52	N/A	2.65 ± 1.26	Day 7 2.35 ± 0.95 Day 30 **1.8 ± 0.7** *	1***
	Control	33	15.71 ± 7.36	Day 7 18.20 ± 8.24 Day 30 15.6 ± 8.13	12.96 ± 5.05	Day 7 10.5 ± 4.89 Day 30 12.95 ± 4.5	42.65 ± 25.2	N/A	3 ± 1.3	Day 7 3.1 ± 1.44 Day 30 2.8 ± 1.15	3
Chung (2015)	Tamsulosin	44	13.2 ± 7.4	Day 7 12.5 ± 8.3	12.1 ± 6.4	Day 1 **15.7 ± 8.3** * Day 7 **13.7 ± 7.6** *	40.7 ± 33.0	Day 1 **39.7 ± 25.3** * Day 7 38.5 ± 18.5	3.4 ± 1.1	Day 7 3.2 ± 1.1	0***
	Control	44	13.5 ± 8.6	Day 7 12.8 ± 8.7	13.0 ± 4.4	Day 1 12.9 ± 4.8 Day 7 11.4 ± 4.3	53.8 ± 43.9	Day 1 78.8 ± 75.2 Day 7 67.1 ± 116.7	3.4 ± 1.2	Day 7 3.2 ± 1.3	2
Efesoy (2021)	Alfuzosin	24	13.20 ± 3.72	Day 7 **9.41 ± 2.41** * Day 30 **8.79 ± 2.02** *	12.87 ± 3.09	Day 7 **14.25 ± 3.19** * Day 30 **15.45 ± 2.97** *	52.33 ± 18.07	Day 7 **32.41 ± 11.85** * Day 30 **30.66 ± 11.96** *	2.58 ± 1.05	Day 7 **1.29 ± 0.69** * Day 30 **1.12 ± 0.67** *	0***
	Doxazosin	25	12.68 ± 4.12	Day 7 **9.32 ± 2.24** * Day 30 **8.56** **± 2.51** *	12.40 ± 2.54	Day 7 **14.04 ± 3.42** * Day 30 **15.16 ± 3.35** *	49.24 ± 17.55	Day 7 **29.88 ± 12.85** * Day 30 **26.28 ± 12.26** *	2.32 ± 1.06	Day 7 **1.24 ± 0.77** * Day 30 **1.04 ± 0.73** *	0***
	Silodosin	23	13.31 ± 3.69	Day 7 **8.73 ± 3.41** * Day 30 **7.86 ± 2.94** *	12.17 ± 2.28	Day 7 **14.43 ± 2.82** * Day 30 **15.60 ± 3.58** *	48.95 ± 16.14	Day 7 **34.17 ± 14.19** * Day 30 **31.00 ± 13.38** *	2.43 ± 0.84	Day 7 **1.21 ± 0.42** * Day 30 **1.08 ± 0.51** *	0***
	Tamsulosin	26	12.15 ± 3.96	Day 7 **8.23 ± 2.80** * Day 30 **7.73 ± 1.99** *	13.07 ± 2.26	Day 7 **14.96 ± 2.42** * Day 30 **16.15 ± 1.75** *	55.53 ± 14.94	Day 7 **35.30 ± 13.26** * Day 30 **31.50 ± 16.29** *	2.42 ± 1.06	Day 7 **1.19 ± 0.63** * Day 30 **1.03 ± 0.59** *	0***
	Terazosin	23	12.82 ± 3.58	Day 7 **9.13 ± 2.36** * Day 30 **8.30 ± 1.94** *	12.73 ± 2.43	Day 7 **14.26 ± 2.41** * Day 30 **15.34 ± 2.10** *	52.95 ± 17.48	Day 7 **34.39 ± 12.17** * Day 30 **32.26 ± 11.87** *	2.13 ± 0.91	Day 7 **1.21 ± 0.79** * Day 30 **1.00 ± 0.67** *	0***
	Control	25	12.60 ± 4.84	Day 7 15.32 ± 4.08 Day 30 12.64 ± 4.48	12.57 ± 2.55	Day 7 10.68 ± 1.74 Day 30 12.12 ± 2.14	54.67 ± 16.99	Day 7 66.24 ± 19.91 Day 30 55.32 ± 16.85	2.21 ± 0.91	Day 7 3.00 ± 1.38 Day 30 2.36 ± 1.35	3
Salar (2022)	Silodosin	99	13.8 ± 8.8	Day 7 12.4 ± 10.5	12.1 ± 6.2	Day 7 **12.3 ± 6.9** *	107.8 ± 70.4	Day 7 **100.3 ± 93.5** *	N/A	N/A	**2** *
	Control	98	12 ± 9.4	Day 7 14.4 ± 10.1	12.6 ± 7.0	Day 7 9.3 ± 6.9	92.1 ± 79.9	Day 7 125.3 ± 63.5	N/A	N/A	9
Sefik (2020)	Tamsulosin	55	13.1 ± 4.9	Day 7 **11.7 ± 4.2** * Day 30 13.6 ± 6.5	14.3 ± 4.4	Day 7 **15.1 ± 4.3** * Day 30 14.3 ± 4.8	36.3 ± 30.3	Day 7 21.3 ± 22.9 Day 30 20.8 ± 23	N/A	Day 30 68 ± 19.3**	0***
	Control	56	12.8 ± 5.2	Day 7 15.5 ± 5.2 Day 30 15.6 ± 5.7	13.8 ± 5.2	Day 7 12.1 ± 5.5 Day 30 13.2 ± 4	25 ± 28.1	Day 7 40.4 ± 63 Day 30 19.8 ± 23.8	N/A	Day 30 61.9 ± 19.2**	1
Zheng (2022)	Doxazosin	100	13.1 ± 0.6	Day 7 **10.1 ± 0.5** * Day 30 **9.4 ± 0.6** *	11.6 ± 0.3	Day 7 **12.6 ± 0.3** * Day 30 **14.0 ± 0.3** *	42.1 ± 2.8	Day 7 39.4 ± 2.7 Day 30 38.7 ± 2.7	2.0 ± 0.1	Day 7 **1.3 ± 0.1** * Day 30 **1.1 ± 0.2** *	2***
	Control	100	12.9 ± 0.5	Day 7 15.8 ± 0.6 Day 30 13.3 ± 0.7	12.2 ± 0.3	Day 7 10.1 ± 0.3 Day 30 11.5 ± 0.2	39.3 ± 2.9	Day 7 40.8 ± 3.2 Day 30 41.6 ± 3.2	2.0 ± 0.2	Day 7 2.4 ± 0.1 Day 30 2.1 ± 0.1	5

*Note*: Values in bold emphasis indicate statistically significant differences (in addition to single asterisks).

* Statistically significant (*p <* 0.05) difference between intervention and control groups.

** Turkish SF‐36 QoL Form. Not statistically significant.

*** *p*‐value not provided.

### 3.5 Maximum voiding flow rate (Qmax)

All six RCTs resulted in higher Qmax values for alpha blocker groups at days 1, 7 and 30 (Table 3). Results were statistically significant except for one study's day 30 measurement.^9^ All day 7 measurements demonstrated statistical significant differences between groups. Pooled analysis demonstrated statistically significantly higher Qmax values (mean difference 3.07, 95% CI: 2.55–3.59) in alpha blocker groups with low heterogeneity (*I* ^2^ = 0%) (Figure 3).

### 3.6 PVR

All five studies that examined PVRs reported lower values in alpha blocker groups at days 1, 7 and 30 except for one study's day 30 measurement (20.8 vs. 19.8, *p =* 0.826)^18^ (Table 3). Only two studies^8^, ^17^ demonstrated statistically significant differences in PVRs for days 1 and 7, respectively. Other differences were not reported to be statistically significant. Pooled analysis showed statistically significantly lower PVRs (mean difference 20.41, 95% CI: 2.44–37.39) in alpha blocker groups with high heterogeneity (*I* ^2^ = 86%) (Figure 3).

### 3.7 QoL index

All five studies resulted in improved QoL indices for alpha blocker groups compared to controls (Table 3). Results were statistically significant in three studies.^3^, ^18^, ^19^ Pooled results further demonstrate this with statistically significantly improved QoL scales (standardised mean difference 0.87, 95% CI: 0.17–1.48) with high heterogeneity (*I* ^2^ = 86%) (Figure 3).

### 3.8 AUR

In all six studies, there were lower incidences of AUR in the alpha blocker groups compared to controls (Table 3). This was statistically significant in one study (2.0% vs. 9.2%, *p =* 0.02).^8^ When pooled, the total incidence between groups were 5 (1.1%) and 23 (6.5%), respectively (*p =* 0.001). Pooled analysis showed significantly lower risk of AUR with an odds ratio of 0.22 (95% CI 0.09–0.55) in alpha blocker groups with low heterogeneity (*I* ^2^ = 0%) (Figure 3).

### 3.9 Side effects of alpha blocker usage

None of the included studies reported significant adverse outcomes related to alpha blocker usage. Two trials^3^, ^17^ reported no side effects as a result of alpha blocker usage, three trials^9^, ^18^, ^19^ did not comment on side effects and one trial^8^ demonstrated similar low rates of vasovagal syncope between both groups (*p =* 0.31).

### 3.10 Meta‐regression and funnel plot analysis

Meta‐regression of age, PSA, PV and alpha blocker categories between studies did not demonstrate any statistically significant confounding variables. Funnel plot analysis and Egger's test for small study effects demonstrated low risk of publication bias across all outcome measures (supporting information Figure S1).

## 4 DISCUSSION

To our knowledge, this study represents the first systematic review and meta‐analysis that has directly investigated the evidence regarding the effect of alpha blockers in reducing postprostatic biopsy voiding dysfunction. This study identified improved voiding function in all objective and subjective voiding outcome measures, as well as reduced incidence of adverse outcomes including AUR.

There is a reported incidence of voiding dysfunction of up to 51.5% post‐TRUS‐guided biopsy,^1^, ^2^, ^3^, ^4^, ^5^ possibly related to prostatic inflammatory oedema, urethral blood clots, sphincteric spasms or postoperative pain.^22^ Perioperative alpha blockers had an overall positive effect on both subjective (IPSS and QoLs scores) and objective (higher Qmax, lower PVRs and lower rates of AUR) voiding function. Additionally, alpha blockers were not reported to have a significant increase in adverse events including hypotension and falls across included studies.

The greatest effect sizes were observed when comparing measurements on day 7 postbiopsy, rather than day 1 or day 30. This may be explained by an inadequate duration of therapy for medications to take effect by day 1 and resolving voiding defects from prostate biopsy by day 30. These results potentially suggest that maximal benefit from therapy is noted within the first 2 weeks postbiopsy. They may also suggest that earlier pretreatment with alpha blockers prior to prostate biopsy may produce more significant improvements in voiding function in the earlier stages postbiopsy such as day 1 post‐op, as majority of included studies only commenced alpha blocker therapy the day prior to biopsy.^3^, ^17^, ^18^, ^19^ Further studies are required to characterise specific subgroups including age, PV and degree of pre‐operative voiding function to identify patients who are likely to benefit most from perioperative alpha blocker usage.

In this review, all studies included patients undergoing TRUS biopsies only. A meta‐analysis demonstrated similar rates of voiding dysfunction or postbiopsy urinary retention between patients undergoing prostate biopsies via TP and TRUS approaches,^23^ with the incidence of postbiopsy voiding dysfunction related largely to prostate size. The rates of AUR in our included studies were 2.8% in keeping with the reported rates in the literature. Unfortunately, there are no studies directly investigating the effects of alpha blockers on TP biopsies. Despite this, based on their mechanism of action, as well as similarly reported rates of AUR, it could be extrapolated that similar effects are likely to be seen in patients undergoing TP biopsies as well.

A strength of this review stems from the inclusion of high quality RCTs only. All trials excluded patients with medical conditions that may confound voiding function results such as Diabetes Mellitus, compared alpha blocker groups to controls, utilised TRUS biopsies, measured the same or similar outcomes (IPSS, PVR, Qmax, AUR, QoL), at similar intervals. Additionally, all articles measured voiding function at day 7 postbiopsy, which allowed for pooling in a meta‐analysis. Despite this, there is noted variability in the specific alpha blocker used and duration of therapy. Differences in PV were not noted to be significant between included articles except for Efesoy 2021. This is important, as increased PV may lead to a greater likelihood of pre‐operative voiding impairment and predisposes to postoperative voiding impairment in the absence of known issues.^24^

The varied usage, dose and duration of alpha blockers among the reviewed publications are a limitation of our study. This likely contributed to significant heterogeneity as demonstrated by the moderate to high *I* ^2^ statistics. Another previously discussed limitation is the absence of trials investigating TP biopsies. This review and its positive findings are directly applicable to TRUS biopsies, and similar outcomes may be observed with the TP approach. However, further studies are needed to characterise the effect size and significance of this.

### 4.1 Perspectives

Various health services already incorporate the usage of perioperative alpha blockers to prevent post‐biopsy voiding dysfunction. A few examples include West Suffolk NHS Foundation Trust in the United Kingdom as well as Western Health and Fiona Stanley Hospital in Australia.^10^, ^11^ Despite this, there has been no published systematic review pooling and formally assessing the evidence of this in the literature. This review has implications for clinical practice by suggesting the routine use of alpha blocker therapy in patients undergoing prostate biopsy and potentially standard of care. Incorporating this into clinical guidelines may lead to better patient outcomes and satisfaction by improving voiding function and significantly reducing the risk of voiding complications.

## 5 CONCLUSION

These systematic review and meta‐analysis have demonstrated that efficacy of perioperative alpha blockers in managing voiding dysfunction secondary to TRUS biopsy, particularly within the first week post‐operatively. These findings have important implications for clinical practice, supporting their routine usage to improve patient outcomes and satisfaction. Further research is warranted to validate their efficacy, refine treatment protocols and evaluate their effect on TP biopsy.

## AUTHOR CONTRIBUTIONS

**Sean Lim**: Conceptualisation, Screening of abstracts/Full texts, quality assessment, data collection, manuscript writing, primary driver of study. **Kylie Yen‐Yi Lim**: Screening of abstracts/Full texts, quality assessment, data collection. **Liang Qu**: Conceptualisation, Manuscript writing. **Sanjeeva Ranasinha**: Statistical Analysis. **Anthony Dat**: Manuscript writing. **Matthew Brown**: Manuscript writing. **Paul Manohar**: Manuscript writing. **Matthew Harper**: Manuscript writing. **Scott Donnellan**: Manuscript writing. **Weranja Ranasinghe**: Conceptualisation, Supervising author, Manuscript writing.

## CONFLICT OF INTEREST STATEMENT

All authors report no conflicts of interest.

## Supporting information


**Figure S1:** Funnel plots with 95% confidence intervals

**Table S1.** Search Strategy
