# Supplementary material for: Perioperative alpha blockers in voiding dysfunction secondary to prostate biopsy: A meta‐analysis
Source: BJUI Compass. 2024 May 8;5(8):748–60. doi: 10.1002/bco2.366 (PMC11327493; doi:10.1002/bco2.366)
Supplement: Supplementary file 1 — Figure S1: Funnel plots with 95% confidence intervals [file BCO2-5-748-s001.docx]

**Supplementary Figure 1: Funnel plots with 95% confidence intervals**

**B)**

**A)**

**C)**

**D)**

**E)**

A) AUR, p=0.402; B) IPSS, p=0.131; C) PVR, p=0.131; D) Qmax, p=0.865; E) QoL Index, p=0.911
