# Supplementary material for: Perioperative alpha blockers in voiding dysfunction secondary to prostate biopsy: A meta‐analysis
Source: BJUI Compass. 2024 May 8;5(8):748–60. doi: 10.1002/bco2.366 (PMC11327493; doi:10.1002/bco2.366)
Supplement: Supplementary file 2 — Table S1. Search Strategy [file BCO2-5-748-s002.docx]

**Search Strategy**

| **#** | **Query** |
| --- | --- |
| **1** | (alpha blocker* or alpha-blocker* or alpha receptor blocker*).ti,ab. |
| **2** | (5alpha reductase or 5-alpha reductase or 5-alpha-reductase).ti,ab. or Cholestenone 5 alpha-Reductase/ |
| **3** | tamsulosin.ti,ab. or Tamsulosin/ |
| **4** | solifenacin.ti,ab. or Solifenacin Succinate/ |
| **5** | prazosin.ti,ab. or Prazosin/ |
| **6** | doxazosin.ti,ab. or Doxazosin/ |
| **7** | Prostatic Neoplasms/ or Prostate/ |
| **8** | (transperineal biops* or transrectal biops*).ti,ab. |
| **9** | prosta*.ti,ab. or Prostatic Neoplasms/ or Prostate/ |
| **10** | (biops* or transperineal biops* or transrectal biops*).ti,ab. |
| **11** | Biopsy, Needle/ |
| **12** | 1 or 2 or 3 or 4 or 5 or 6 |
| **13** | 10 or 11 |
| **14** | 9 and 12 and 13 |
